# Association of Assisted Reproductive Technology Treatments with Imprinting Disorders

**DOI:** 10.1055/s-0041-1723085

**Published:** 2021-02-25

**Authors:** T. Kopca, Pinar Tulay

**Affiliations:** 1 Department of Medical Genetics, Faculty of Medicine, Near East University, Nicosia, Cyprus; 2 Near East University, DESAM Institute, Nicosia, Cyprus

**Keywords:** assisted reproductive technology, imprinting disorders, IVF, epigenetics

## Abstract

Assisted reproductive technology (ART) is a broad field in infertility that encompasses different types of treatments. These revolutionary treatment methods aimed to aid infertile or subfertile couples. Treatment was expanded exponentially, as 1 to 3% of the births worldwide takes place with ART procedures. However, treatment is not flawless. Gametes and embryos are exposed to different chemicals and stress through treatment, which leads to disturbance in proper embryo development and results in prenatal and congenital anomalies. When compared with in-vivo development of gametes and preimplantation embryos in mice, in-vitro conditions during ART treatments have been suggested to disturb the gene expression levels, especially imprinted genes. Therefore, ART has been suggested to be associated with increased incidences of different imprinting disorders such as Beckwith–Wiedemann syndrome, Angelman syndrome, and Silver–Russell syndrome, as proved by different case reports and studies. This literature review aims to explain the association of imprinting disorders with this revolutionary treatment procedure.

## Introduction

Infertility is a health condition in which individuals fail to achieve a pregnancy following unprotected sexual intercourse within a year. 1 Both females and males can suffer from infertility. It affects ∼15% of individuals who are in their reproductive ages worldwide. 2 Genetic causes such as chromosomal aberrations or mutations, environmental factors that lead to reduction in the quality of germ cells, and defects in reproductive organs are the main reasons behind the incapacity to fulfill pregnancy in couples. Until 1978, couples who suffered from infertility had no option to achieve pregnancy and have a biological child. In 1978, a new therapeutic approach was born—called in-vitro fertilization (IVF). 1 This procedure consists of a series of complex stages that ends up with the fusion of collected sperm and oocyte in laboratory conditions and culturing of the zygote. The cultured embryo is transplanted to the female uterus. Over the 40 years, advances in the field of assisted reproductive technology (ART) have been made by a team comprising gynecologists, embryologists, and geneticists to elevate success rate and increase accessibility for the patients. ART has become a powerful therapeutic approach for infertile couples who want to have a biological child. While ART was proposed to be a safe and powerful approach in the beginning, subsequently, it has suggested to be associated with negative outcomes. Several studies have shown that children who were conceived by ART show a high predisposition to different disorders such as heart malformations, autism spectrum disorders, tumorigenesis, and diabetes. 3 Besides that, ART-conceived children show high incidence of somatic epigenetic alterations and also distortions in the imprinting genes that can normally lead to rare imprinting disorders.

### Preimplantation Embryo Development

Preimplantation embryonic development in mammals consists of several significant steps. These steps start from gametogenesis and progress until the delivery of the fetus. As a result of spermatogenesis and oogenesis, respectively, female and male gametes are derived in the form of primordial germ cells (PGCs). When the mitosis starts in the PGCs in mice, the formation of the germ cells occurs differently in each gender.

For spermatogenesis in males, spermatogonia undergoes mitosis from puberty until death of the individual. During the process, four spermatids are generated from each spermatocyte at the end of the meiosis. But in oogenesis, differentiation in female mouse PGCs leads to the formation of oogonia and then they undergo meiosis. During oogenesis, all oocytes are arrested during prophase I in the ovary until puberty. At puberty, with a hormonal surge, oocyte completes meiosis I and after the formation of a secondary oocyte, it arrests at metaphase II. In metaphase II, transcription is terminated and the rate of messenger ribonucleic acid (RNA) translation is diminished. 4 Meiosis II is completed after the fertilization and each oocyte produces I functional oocyte that becomes the maternal pronucleus. 5

After the fertilization, fusion of female and male pronuclei leads to formation of syngamy. 6 Mammalian embryo in the one-cell stage consists of both paternal and maternal haploid pronuclei that are provided with sperm and oocyte. Pronucleus of each parent is replicated before mitosis. Two-cell embryo formed by first cleavage division consists of two diploid cells that contain one set of paternal and one set of maternal chromosomes. For initiation of the embryonic development and embryonic genome activation (EGA), maternal and paternal chromosomes are programmed in the embryonic genome during the cleavage stage divisions. Embryonic genome is activated in two-cell stage and four- to eight-cell stage in mouse and human embryos, respectively. 7 The degradation of the maternal nucleic acids, proteins, macromolecules, and specific RNAs stored in oocytes leads to the initiation of this activation process. 8 After EGA, reprogramming occurs saliently in the preimplantation embryo. Maintenance of these programming events is made possible with the help of epigenetic controlling mechanisms such as deoxyribonucleic acid (DNA) methylation, histone acetylation, as well as with small noncoding RNAs, microRNAs, and transcription and translation processes.

### Epigenetic Reprogramming after Fertilization

The term epigenetics refers to heritable and stable changes in the DNA sequence that affect phenotype by alteration at the expression of a gene, that do not involve changes at DNA sequence. 9 In human embryonic development, epigenetic alterations are responsible for the determination of cell fate that leads to differentiation of the cells into distinct functions. DNA methylation is one of the important processes in human embryonic development. Addition of methyl groups to the DNA sequences leads to silencing of gene expression and acts as a restriction barrier at different stages in mammalian development. These methylation marks, in other words, will be annihilated at different phases of development when developmental potency needs to be altered. At the beginning of the process, maternal and paternal pronuclei are hypermethylated. Epigenetic barriers are demolished first time right after the fertilization for determination of the cell function, for which it is responsible in the future, and for changing totipotency. Demethylation occurs in two stages. The first annihilation procedure takes place at paternal pronucleus and drives it in the rapid demethylation process. This is followed by loss of methylation patterns at maternal pronucleus in the developing preimplantation embryo. Subsequently, DNA methylation patterns are constituted again when the developing embryo is at the blastocyst stage. This process gives rise to the formation of epiblast, which is developmentally constricted. PGCs inherit the DNA methylation patterns of the developmentally restricted epiblast cells. Abstersion of the methylation marks takes place again after the generation of the PGCs and occurs at an extensive level for the reconstruction of potency level, which is called global scale demethylation. 10 11 Different studies suggest that ART is involved in the manipulation of embryonic development by gamete stimulation. Also, these manipulations affect gene expression by alteration in the epigenetic controlling mechanisms.

### Imprinting

Imprinting genes are set of genes that are differentially expressed and their expression determined parentally that contributed them. This means when an allele is paternally imprinted, the maternal allele is expressed in child's genotype and phenotype. Genomic imprinting occurs with modifications in nucleotides chemically. This leads to remaining of one functional and one silenced allele at the specific gene region. These genes have an important role in the fetal development. Imprinting genes are in a perfect balance by the virtue of epigenetic mechanism. Imprinting at the specific gene regions is a dynamic process and is conducted with two major epigenetic mechanisms, which are DNA methylation and histone modifications. DNA methylation process occurs as a result of binding methyl groups at cytosine residues of Cytosine phosphate guanine (CpG) islands. 12 These dinucleotides consist of a major part of the promoter regions of the human genome. Promoter regions are methylated through an enzyme called DNA methyltransferase 1 (DNMT1). DNMT1 is a sequence-independent methyltransferase enzyme that can make a hemi-methylation on the gene. As a result of hemi-methylated genome, DNA methylation status is maintained during the replication and results in the formation of proper methylation pattern in cell divisions. However, there are other groups of enzymes called DNMT3A and DNMT3B, which are methyltransferases with specific features. These group of enzymes can make de novo methylation at the early developmental stage and specific stages of gametogenesis. Thus, they assist in the formation of methylation patterns according to the parent of origin. 13

### Manipulations of ART

According to the experimental shreds of evidence, ART may have an effect on the embryonic development in different ways. The manipulations of hormones used for downregulation of the pituitary gland function and for enhancing supernumerary oocyte production by exogenous hormones to retrieve multiple oocytes for increasing the success rate of the therapy is one of the factors that affect the oocyte development. Also, the application of the immature sperm for intracytoplasmic sperm injection (ICSI) may lead to alterations in embryo development. 14

## Different Procedures of ART That Can Affect Preimplantation Embryo, and Pre and Postnatal Development

### Ovarian Hyperstimulation

Ovarian hyperstimulation procedure plays an important role in the compensation of the inefficiencies that could occur in IVF. Treatment takes place with the administration of high doses of gonadotropins to downregulate pituitary function and promote the ovulation to produce multiple oocytes in one cycle. Retrieval of multiple oocytes increases the success rate of therapy by the selection of one or more embryos for transfer or cryopreservation of selected embryos for later cycles. Nevertheless, administration of the exogenous gonadotropins leads to detrimental effects on embryos by altering epigenetic modification mechanism. Spontaneously derived oocytes complete their primary imprinting process at the late stage of the oogenesis. Utilization of gonadotropins for superovulation may alter imprinting attainment of mature oocytes. Retrieved oocytes from superovulation might be released prematurely without completing the imprinting process, or ovulation without treatment can lead to the maturation of poor-quality oocytes. 15

### IVF Culture

Postfertilization culture has a critical significance for the correct development and preimplantation embryos. Proper culture must contain all nutritional components that an embryo needs to grow, such as proteins, amino acids, and energy substrates. Over the 40 years, efficiency of culture media has improved. However, there are extensive shreds of evidence according to studies that use of different culture media and different culture conditions leads to different disorders. Culture media and its conditions can show variations from species to species and even between different laboratories. Cultures are diversified according to energy components, ingredients, and oxygen tensions. Especially, different embryo cultures and their additives such as glucose, serum, and amino acids lead to significant differences in the gene expression patterns and imprinting profiles according to mouse embryo culture studies.

There are many studies executed for the observation of the impact of different culture media on embryos. Mostly mouse embryos were used because they provide a good model to understand the mechanism of human genomic imprinting with their genome similarities.

One of the studies demonstrated the differential expression of H19 imprinting gene in two different culture media in mice. It is highly active in different tissues in prenatal period and has an important role in the embryonic development. In in-vivo conditions, H19 gene imprinting control region is hypermethylated in the paternal genome while it has maternal specific expression. *Whitten's* medium and potassium simplex optimization medium with amino acids (KSOM ^AA^ ) were used in this study. 16 17 Whitten's medium is one of the first used media types for murine models, with the composition of Krebs Ringer bicarbonate, glucose, streptomycin, penicillin G, and bovine serum albumin. But KSOM ^AA^ was generated with increased concentration of potassium chloride and sodium chloride. 18 Also, the efficiency of the media was improved with the addition of 19 nonessential amino acids. In *Whitten's* medium, normally silenced paternal H19 gene was expressed aberrantly in two-cell stage mouse embryos and resulted with biallelic expression. However, embryos that were cultured in KSOM ^AA^ media have demonstrated convenient methylation patterns with in-vivo conditions. As a result of the study, loss of methylation was observed in CpG islands of imprinting control regions at Whitten's medium derived embryos, which means that the gene shows adverse response to this medium. However, embryos better adapted to KSOM+ ^AA^ culture than Whitten's medium because it simulates more closely the in-vivo conditions.

Another study with mouse embryos has demonstrated different expression levels of imprinting genes, including Igf2, H19, Grb7, and Grb10, at early developmental stage. The study was performed in two-step culture media, which were both M16, but one of them with fetal calf serum (FCS). M16 + FCS derived fetuses were shown to have lower expression levels of H19 and Igf2 imprinting genes related with the increased rate of DNA methylation at the imprinting control regions of H19. Also, they observed that the expression levels of Grb10 imprinting gene was increased while Grb7 rate was decreased in the M16 + FCS fetuses. 19

To date, there are no well-established mechanisms investigating how different culture media alter gene expression and lead to imprinting disorders in preimplantation embryos, though there are some plausible hypotheses found to understand these genetic alterations. One of the possibilities is that, in in-vitro conditions, temporal movement of the DNMT1o or related protein to the nucleus is altered. DNMT1o has a role in the maintenance of the imprinting patterns during global DNA demethylation in the preimplantation embryos. Time-dependent DNMT1o must be translocated into the nucleus after the fertilization, not in the embryo cleavage. Because of the retardation of embryonic cleavage in in-vitro conditions, translocation of the DNMT1 to the nucleus could occur in the wrong developmental stage. Due to similarities between the mouse and human embryos, human imprinting patterns could be affected in similar ways following use of different culture media in ART procedures. Another possibility is the alteration or disruption of factors that have a role in the maintenance of imprinting patterns such as DNMTs or related proteins, or alteration of the chromatin structure due to stress in in-vitro culture. Stress leads to modifications in chromatin structure and affects the imprinting patterns even though there are proper mechanisms found for maintenance of imprinting patterns. Also, one of the assumptions defends that proteins, components, or serum that are added into the culture media to provide fetal development seem to affect the development of preimplantation embryos. Even though it is not known which of them can affect unfavorably, it is thought to be related with alteration of cell cycle kinetics. Affected cell cycle kinetics related with the alteration in the imprinting maintenance mechanisms, such as the H19, upstream in differentially methylated regions (DMRs) in subsequent cell cycles. These mechanisms are not only responsible for propagation of the DNA methylation of one of the parental alleles, but also they are most likely associated with the maintenance of specialized chromatin features and nonhistone proteins at other alleles. As a consequence of serum-induced alterations, cell cycle kinetics will be changed in the early developmental stages and can lead to deterioration of proper imprinting maintenance mechanism. 20 21 22 23

### Intracytoplasmic Sperm Injection

ICSI is widely used in ART for men who have suboptimal sperm quality or sperm count. The procedure involves the injection of deficient mobility or abnormal morphology spermatozoa directly into the oocyte. However, utilization of abnormal morphology and impaired motility sperms in treatment leads to an increase in the incidence of different disorders in the offspring due to the quality of gametes. Studies have shown that males who suffer from moderate oligospermia and severe oligospermia are associated with increased incidence to altered methylation profile at H19 gene. Further studies investigated the methylation patterns at imprinting regions between males who had normozoospermia and had abnormal semen counts. Study presented that men who had normal semen counts completed methylation establishment successfully. However, abnormal sperms that were collected for ICSI demonstrated that they were adversely affected from improperly established methylation patterns at imprinting regions. As a result of the studies, this method increases the potential of epigenetic modifications and leads to conceiving embryos with Angelman syndrome (AS), Silver–Russell syndrome (SRS), or Beckwith–Wiedemann syndrome (BWS). 24 25 26

### Implications of ART on Imprinting Disorders

As mentioned previously, significant numbers of studies are proving the relationship between ART-conceived children and increased risk of imprinting disorders. 25 Imprinting disorders are caused by different mechanisms such as mutation or deletion at specific imprinting genes, deletion or duplication that encompasses imprinting genes, and uniparental disomy. ART has especially been associated with BWS, AS, and SRS. 27

#### Beckwith–Wiedemann Syndrome

BWS is an imprinting disorder that is caused by mutations, deletions, or epigenetic alterations leading to disturbance in regulation of the specific genes on the chromosome 11p15.5. 28 Symptoms of the syndrome vary among individuals. The common symptoms of the syndrome include macrosomia and omphalocele. 29 Moreover, infants who suffer from BWS are prone to develop embryonal tumors such as Wilms tumor. Normally, paternal imprinting region contains IGF2 and KCNQ1OT1 imprinting genes, whereas H19, CDKN1C, and KCNQ1 genes are expressed from the maternal allele. 28 Most of the cases are sporadic and occur due to the epigenetic changes rather than genetic changes. In up to 60% of the cases, epigenetic alterations at DMRs lead to aberrant expression or methylation of paternal or maternal genes within chromosome 11. For instance, loss of maternal allele methylation at DMR 1 (kvDMR1) between CDKN1C and IGF2 genes is associated with loss of CDKN1C growth suppressor gene and results in overgrowth. 30 Several studies have been performed for establishing the association between BWS and ART. One of the studies showed that seven children born with BWS were all conceived by ART procedures and had no family history of the disorder. One of these children was conceived using donor eggs. DNA samples from affected children demonstrated that five of six children had abnormal imprinting at LIT1 gene due to hypomethylation of the DMR of the LIT1. One of them also indicated the hypomethylation at H19 DMR. Only one of the children showed a normal methylation pattern in both LIT1 and H19. 31 Similar associations of abnormal methylation profiles and imprinted genes were shown to lead to birth of children with BWS following ART procedures.

### Angelman Syndrome

AS is a rare complex disorder that is characterized by severe mental retardation, ataxia, and speech impairments. 32 Cases of the AS are caused by loss of function of the UBE3A gene. The gene is located on chromosome 15q11.2. UBE3A is inherited from each parental allele and both copies of them are found in cells, but only maternal copy of the gene is active in the cells. Syndrome is generally caused by deletion of UBE3A maternal copy in the cells. 33 Case reports that were performed on AS patients have shown that subfertile couples who were treated with ICSI or ovarian hyperstimulation at the time of pregnancy had twice as much increased risk to have a child with AS or other imprinting disorders when compared with subfertile couples without treatment at the time of pregnancy. Multiple studies also gathered under the same discussion about the association of increased prevalence of AS with utilization of defective sperm samples or ovarian stimulation. 24 34

### Silver–Russell Syndrome

SRS is a condition characterized by low birth weight and slow growth at the postnatal period. Patients who suffer from the syndrome have difficulties to gain weight at a proper rate with normal head circumference. 35 Patients have triangular face structure, prominent head, and small jaw. It is a complex disorder and caused by the aberration of genes, located on chromosome 7 and 11, 36 that have a role at controlling growth similar to the previously described disorders, ART and SRS was associated with. SRS suspected features with no major abnormalities in a child born in a twin pregnancy and obtained from ART treatments were reported. Different methylation patterns (partial hypermethylation) were observed at PEG1/MEST DMR with no significant difference in the H19 and SNRPN DMR regions in this child. 37 Also, hypermethylation at the paternal gene was detected and this was an indication for subfertility. 38 According to the murine models, PEG1/MEST knockout mice inherited paternal genes that led to growth retardation. 39 Study indicates that enhanced methylation rates are the result of ART, which provides a genetic mechanism for alteration in methylation patterns and leads to low birth weight due to the utilization of improper sperm samples and IVF environmental factors such as culture media components. 37

## Conclusion

In conclusion, ART-conceived embryos have been shown to have an increased incidence of various imprinting disorders due to the genetic and epigenetic variations during embryonic development. Different phases of ART, such as ovarian stimulation, IVF, ICSI, and culturing conditions, may affect the most important period of epigenetic reprogramming. Besides, utilization of poor-quality or immature oocytes and abnormal sperm samples results in failure at reprogramming. As a consequence of alterations at epigenetic reprogramming, embryos are conceived with altered methylation patterns and result in aberrant imprinting patterns that lead to rare imprinting disorders.
